# Supplementary material for: D-Lactic Acid Production from Sugarcane Bagasse by Genetically Engineered Saccharomyces cerevisiae
Source: J Fungi (Basel). 2022 Aug 3;8(8):816. doi: 10.3390/jof8080816 (PMC9410322; doi:10.3390/jof8080816)
Supplement: Supplementary file 1 [file jof-08-00816-s001.zip › jof-1782000-supplementary.pdf]

Supporting Information for

D-lactic acid production from sugarcane bagasse by genetically engineered *Saccharomyces cerevisiae*

Authors

Warasirin Sornlek [1,2], Kittapong Sae-Tang [1], Akaraphol Watcharawipas [3], Sriwan Wongwisansri [1], Sutipa Tanapongpipat [1], Lily Eurwilaichtr [4], Verawat Champreda [1],*, Weerawat Runguphan [1], Peter J. Schaap [2] and Vitor A.P. Martins dos Santos [2,5,6*]

Affiliation

[1] National Center for Genetic Engineering and Biotechnology (BIOTEC), 113 Thailand Science Park, Phahonyothin Road, Pathum Thani 12120, Thailand; warasirin@biotec.or.th (W.S.); kittapong@biotec.or.th (K.S.-T.); sriwan@biotec.or.th (S.W.); sutipa@biotec.or.th (S.T.); verawat@biotec.or.th (V.C.); weerawat.run@biotec.or.th (W.R.)

[2] The Laboratory of Systems and Synthetic Biology, Wageningen University and Research, Stippeneng 4,
6708 WE Wageningen, The Netherlands; peter.schaap@wur.nl (P.J.C.); warasirin@biotec.or.th (W.S.)

[3] Department of Microbiology, Faculty of Science, Mahidol University, 272 Rama VI Road, Ratchathewi, Bangkok 10400, Thailand; akaraphol.wat@mahidol.ac.th

[4] National Science and Technology Development Agency, 111 Thailand Science Park, Phahonyothin Road, Pathum Thani 12120, Thailand; lily@nstda.or.th (L.E.)

[5] Laboratory of Bioprocess Engineering, Droevendaalsesteeg 1 6708 PB Wageningen, The Netherlands; vi-tor.martinsdossantos@wur.nl (V.A.P.M.d.S.)

[6] LifeGlimmer GmbH, Markelstrasse, 38, 12163, Berlin, Germany; vitor.martinsdossantos@wur.nl (V.A.P.M.d.S.)

* Correspondence: verawat@biotec.or.th (V.C.); vitor.martinsdossantos@wur.nl (V.A.P.M.d.S.); Tel.: +66-2564-6700 (ext. 3446) (V.C.); +31-317482865 (V.A.P.M.d.S.)

Table S1
Primers used in this study

Primer Name	Sequence (5' to 3')
DeltaUp_TDH3pro_F	ATTAGTATGTAGAAATATAGATTCCATTTTGAGGATTCCTATATCCTCGAGGTACCAGTTTATCATTATCAATACTGCC
TDH3Pro_R	TTTGTTTGTTTATGTGTGTTTATTCG
CYC1ter_F	TCATGTAATTAGTTATGTCACGC
CYC1ter_R	GCAAATTAAAGCCTTCGAGC
*Lm*LDH_F	GTTTCGAATAAACACACATAAACAAACAAAATGAAGATTTTTGCTTATGG
*Lm*LDH_R	AATGTAAGCGTGACATAACTAATTACATGATTAGTATTCAACTGCAATAG
CYC1_*Bam*HI_HIS3pro_F	GTTTTGGGACGCTCGAAGGCTTTAATTTGCGGATCCCGTTTTAAGAGCTTGGTG
DeltaDown_HIS3ter_R	GTTGATAAAGGCTATAATATTAGGTATACAGAATATACTAGAAGTTCTCCGAATTCCATAGATCCGTCGAGTTC
delta_gRNA1_*Hin*dIII_F	ATAAAGCTTTATACTAGAAGTTCTCCTCGGTTTTAGAGCTAGAAATA
gRNA_Rev	ATACTCGAGAAAAAAGCACCG
DeltaUp_F	TGTTGGAATAAAAATCCACTATC
*Lm*LDH_int_seq_R	ATAGCAACTCTACCGATATGACCTG
crRNA_F	TGAAAGTTGGTGCGCATG
crRNA_R	GCTAGGATTCCCTGACTGTCT
tSUP4_F	GTTTTTTATGTCTTATCGTGACGC
pCAS9_R	GATCATTTATCTTTCACTGCGGAG
**Donor DNA primers for gene deletion**	
GPD1_rep_F	TATATTGTACACCCCCCCCCTCCACAAACACAAATATTGATAATATAAAGATTTATTGGA
GPD1_rep_R	CCTCGAAAAAAGTGGGGGAAAGTATGATATGTTATCTTTCTCCAATAAATCTTTATATTA
GPD2_rep_F	AGATTCAATTCTCTTTCCCTTTCCTTTTCCTTCGCTCCCCTTCCTTATCAACACTCTCCC

GPD2_rep_R	GGAAAAAGAGGCAACAGGAAAGATCAGAGGGGGAGGGGGGGGGAGAG TGTTGATAAGGAA
ADH1_rep_F	GCACAATATTTCAAGCTATACCAAGCATACAATCAACTATCTCATATACA GCGAATTTCT
ADH1_rep_R	TTTTTTATAACTTATTTAATAATAAAAATCATAAATCATAAGAAATTCGC TGTATATGAG
**Colony PCR primers for screening of gene deletion**	
GPD1_Up_F	TGGTATTGGCAGTTTCGTAG
GPD1_Down_R	ATACGGACGCCAGATGCT
GPD2_Up_F	TTAGCTTACGGACCTATTGC
GPD2_Down_R	ACGGGCCAAATGCGACAT
ADH1_Up_F	CATTGTTCTCGTTCCCTTTC
ADH1_Down_R	CGATGAAGATAGAGCCCAAC

Table S2

Designing of gene specific crRNA for gene deletion and homologous flanking sequences for homologous recombination with pUDE735

Gene	RGEN Target (5' to 3')	Position	Direction	GC contents (%, w/o PAM)	Out-of-frame Score
*gpd*1	(TTTA)GACAGGAGATAGCTCTGACGTGTGA	427	-	52	77.9
*gpd*2	(TTTA)GACACGAGATGGCCCTTACATGAGG	574	-	56	79.1
*adh*1	(TTTG)GCTTTGGAACTGGAATATCTTTGTA	61	-	36	74.2

Homologous flanking sequences to pUDE735

Upstream of BstBI site	5'TGAAAGTTGGTGCGCATGTTTCGGCGTTCGAAACTTCTCCGCAGTGAAA GATAAATGATC3'
Downstream of BstBI site	5'GTTTTTTATGTCTTATCGTGACGCAGTCCCATGGGCCATTACAAACTCAT GCAGACAGTCAGGGAATCCTAGC3'

Note: Each gene specific crRNA was designed by the CRISPR RGEN tools which utilizes the algorithms described by Bae et al., 2014; Bae, Park, and Kim, 2014; Park, Bae, and Kim, 2015. The crRNAs that have high out-of-frame score (>66) with the appropriate characteristics described by Swiat et al., 2017 were chosen.

Table S3
Carboxylic acids, furfural and hydroxymethyl furfural detected in slurry of alkaline pretreated sugarcane bagasse

	**Slurry fraction (g/L)**
Lactic acid	N.D.
Glycerol	0.26 ± 0.00
Formic acid	N.D.
Acetic acid	3.06 ± 0.15
Levulinic acid	N.D.
Furfural	N.D.
Hydroxymethyl furfural	N.D.

* N.D. indicates that the component was not detected

Table S4
Maximum specific growth rate of all *Saccharomyces cerevisiae* strains used in this study

Strain	YPD μmax*(h^{-1})	YPD + synHT μmax*(h^{-1})
CEN.PK2_DLDHΔ*gpd*	0.061 ± 0.006	0.015 ± 0.001
CEN.PK2_DLDHΔ*gpd*Δ*adh*1	0.036 ± 0.004	0.012 ± 0.001
BCC39850	0.082 ± 0.002	0.029 ± 0.002
CEN.PK2-1C	0.060 ± 0.002	0.016 ± 0.001
CEN.PK2_DLDH	0.066 ± 0.005	0.011 ± 0.001
Hybrid2	0.091 ± 0.004	0.032 ± 0.002
Hybrid35	0.058 ± 0.003	0.026 ± 0.003
Hybrid36	0.068 ± 0.003	0.015 ± 0.001

*Data represent mean values of biological triplicates and error bars indicate standard deviations.

Table S5
Glycerol production by *Saccharomyces cerevisiae* strains used in this study

Strain	Glycerol production (g/L)			
	16h	24h	40h	48h
CEN.PK2-1C	1.35 ± 0.03	1.40 ± 0.04	1.47 ± 0.04	1.50 ± 0.04
BCC39850	3.56 ± 0.07	3.89 ± 0.04	3.98 ± 0.05	4.02 ± 0.05
CEN.PK2_DLDH	1.27 ± 0.06	1.92 ± 0.02	2.05 ± 0.10	2.11 ± 0.04
Hybrid2	1.75 ± 0.06	1.86 ± 0.07	1.81 ± 0.09	1.82 ± 0.09
Hybrid35	2.13 ± 0.03	2.95 ± 0.13	2.79 ± 0.20	2.91 ± 0.17
Hybrid36	1.52 ± 0.06	2.67 ± 0.09	3.27 ± 0.09	3.17 ± 0.10
CEN.PK2_DLDHΔ*gpd*	N.D.	N.D.	N.D.	N.D.
CEN.PK2_DLDHΔ*gpd*Δ*adh1*	N.D.	N.D.	N.D.	N.D.

N.D. indicates that the metabolite was not detected

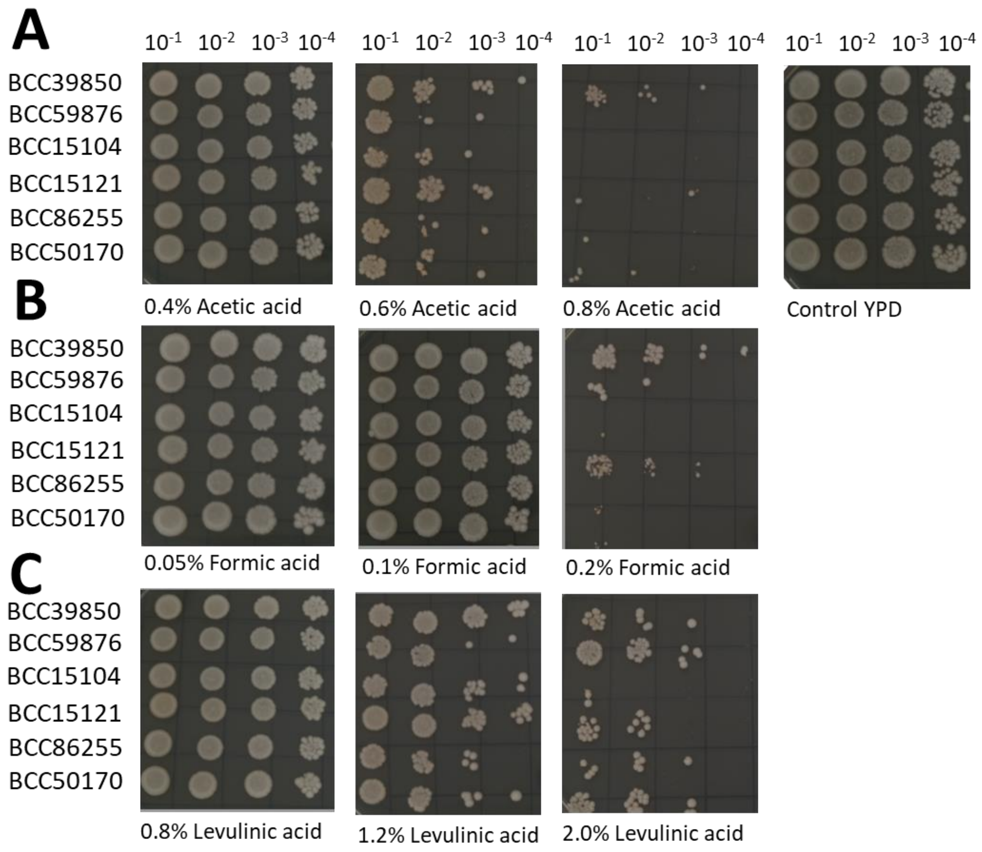

Figure S1 Acetic acid, formic acid and levulinic acid tolerant test of wild *Saccharomyces cerevisiae* strains; BCC39850, BCC59876, BCC15104, BCC15121, BCC86255 and BCC50170. Growth of all strains were recorded at day 3 after spotted on YPD plate containing 0, 4, 6 and 8 g/L acetic acid (**A**), 0.5, 1.0 and 2.0 g/L formic acid (**B**) and 8, 12 and 20 g/L levulinic acid (**C**).

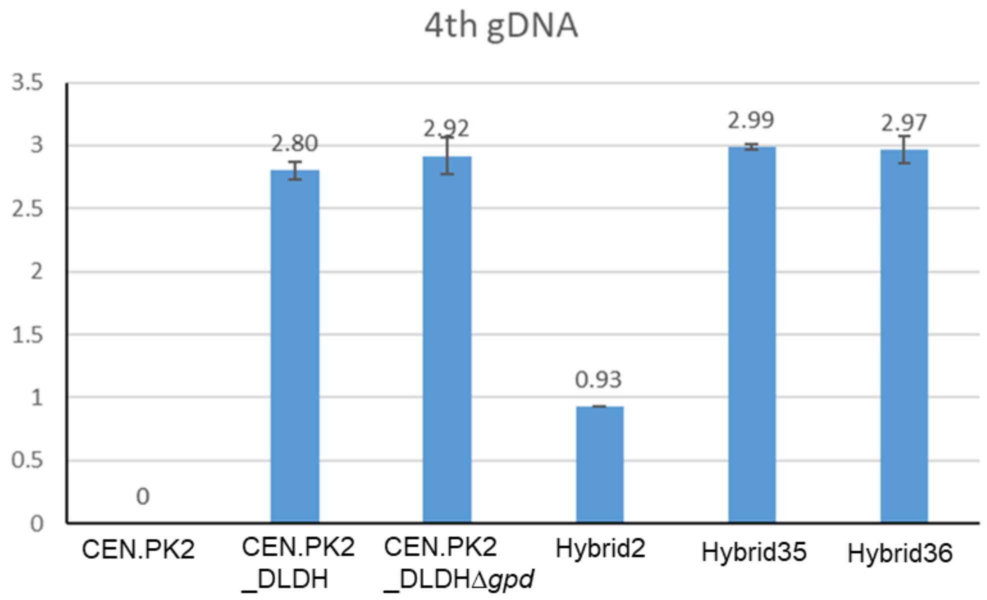

Figure S2 Quantification of *Lm*ldhA copy number among the strains by qPCR
